# TNF receptor 2 knockout mouse had reduced lung cancer growth and schizophrenia-like behavior through a decrease in TrkB-dependent BDNF level

**DOI:** 10.1007/s12272-024-01487-0

**Published:** 2024-04-09

**Authors:** In Jun Yeo, Ji Eun Yu, Sung-Hyun Kim, Dae Hwan Kim, Miran Jo, Dong Ju Son, Jaesuk Yun, Sang-Bae Han, Jin Tae Hong

**Affiliations:** 1 College of Pharmacy and Medical Research Center, Chungbuk National University, 194-31 Osongsaengmyeong 1-ro, Osong-eup, Heungdeok-gu, Cheongju, Chungbuk 28160 Republic of Korea; 2 College of Pharmacy, Kyungpook National University, 80 Daehak-ro, Buk-gu, Daegu, 41566 Republic of Korea; 3 College of Pharmacy, Mokpo National University, 1666, Yeongsan-ro, Muan-gun, Jeonnam 58554 Republic of Korea

**Keywords:** Cancer, Schizophrenia, TNFR2, BDNF, TrkB, TNF-α

## Abstract

The relationship between schizophrenia (SCZ) and cancer development remains controversial. Based on the disease-gene association platform, it has been revealed that tumor necrosis factor receptor (TNFR) could be an important mediatory factor in both cancer and SCZ development. TNF-α also increases the expression of brain-derived neurotrophic factor (BDNF) and tropomyosin receptor kinase B (TrkB) in the development of SCZ and tumor, but the role of TNFR in mediating the association between the two diseases remains unclear. We studied the vital roles of TNFR2 in the progression of tumor and SCZ-like behavior using A549 lung cancer cell xenografted TNFR2 knockout mice. TNFR2 knockout mice showed significantly decreased tumor size and weight as well as schizophrenia-like behaviors compared to wild-type mice. Consistent with the reduced tumor growth and SCZ-like behaviors, the levels of TrkB and BDNF expression were significantly decreased in the lung tumor tissues and pre-frontal cortex of TNFR2 knockout mice. However, intravenous injection of BDNF (160 μg/kg) to TNFR2 knockout mice for 4 weeks increased tumor growth and SCZ-like behaviors as well as TrkB expression. In in vitro study, significantly decreased cell growth and expression of TrkB and BDNF by siTNFR2 transfection were found in A549 lung cancer cells. However, the addition of BDNF (100 ng/ml) into TNFR2 siRNA transfected A549 lung cancer cells recovered cell growth and the expression of TrkB. These results suggest that TNFR2 could be an important factor in mediating the comorbidity between lung tumor growth and SCZ development through increased TrkB-dependent BDNF levels.

**Supplementary Information:**

The online version contains supplementary material available at 10.1007/s12272-024-01487-0.

## Introduction

Several reports have suggested that the development of tumors may be associated with schizophrenia (SCZ) (Shi et al. 2018; Nordentoft et al. 2021). Recent clinical findings have indicated that patients diagnosed with SCZ have an approximately 50% increased risk of death due to breast, lung and colon cancer (Ni et al. 2019). It was also reported that anti-SCZ drugs such as pimozide and penflurdiol have been used for the treatment of tumors including melanoma, lung carcinoma, breast cancer, and glioma (Shaw et al. 2021). Phosphatidylinositol 3-kinase (PI3K), protein kinase B (PKB; also known as AKT), and mechanistic target of rapamycin kinase (mTOR) have been suggested as common signals mediating the development of neurodegenerative diseases such as SCZ, Alzheimer’s disease, Parkinson’s disease, and Huntington’s disease, and the development of cancer including lung, breast, colorectal, and prostate (Vanderplow et al. 2021; Sanaei et al. 2022). In addition, parkin (PARK2), ataxia telangiectasia mutated (ATM), phosphatase and tensin homolog deleted on chromosome 10 (PTEN), and receptor-type tyrosine-protein phosphatase delta (PTPRD) have been demonstrated to be critical targets in the development of cancers and neurodegenerative diseases (Plun-Favreau et al. 2010; Seo and Park 2020). However, clear mechanisms and common targets are not elucidated to date.

Inflammatory cytokines and growth factors such as interleukin-6 (IL-6), interleukin-1β (IL-1β), brain-derived neurotrophic factor (BDNF), and vascular endothelial growth factor (VEGF) have been also demonstrated to play important roles in both the development of cancers and neurodegenerative diseases (Ogunmokun et al. 2021; Sochal et al. 2022). It is noteworthy that tumor necrosis factor alpha (TNF-α) has been reported to play a critical role in some cancers and SCZ development (Pandey et al. 2015; Rodrigues-Amorim et al. 2018; Patlola et al. 2022). TNFRs, which respond to TNF-α are also involved in the development of cancer and SCZ (Hoseth et al. 2017; Martínez-Reza et al. 2017; Qu et al. 2017). However, the role of TNF-α and its receptor TNFRs (TNFR1 and 2) and whether they can act as common targets in the development of cancer and SCZ has not been studied.

In tumorigenesis, TNFR plays important roles in multiple aspects of tumor progression, including the proliferation of cancer cells, the evasion of immune surveillance, the activation of endothelial cells and angiogenesis, and the formation of a pre-metastasis milieu (Sheng et al. 2018; Atretkhany et al. 2020). Especially, TNFR2 converts the tumor inhibiting ability of TNF-α into a tumor advocating factor, thereby directly promoting the proliferation of some types of cancers such as lung, breast, colon, and skin cancer (Sheng et al. 2018). A recent study showed that the transmembrane TNF-α (tmTNFα) is more relevant than soluble TNF-α in the brain of SCZ patients (Pandey et al. 2015). In our previous study (Yeo et al. 2022), we observed increased levels of tmTNF-α in the brain endothelial cells of individuals diagnosed with schizophrenia (SCZ). Furthermore, our investigation revealed that mice with a knockout in TNFR2 and an overexpression of tmTNF-α exhibited an absence of SCZ-like symptoms. In contrast, mice with a knockout in TNFR1 and overexpressing tmTNF-α presented symptoms associated with SCZ. The reported data indicated that TNFR2 could be more significant for SCZ development than TNFR1. Thus, there is possibility of existence of a potential common target for TNFR2 to mediate the relationship between the development of SCZ and cancer. Therefore, we investigated the relationship between the development of lung cancer and SCZ, and whether TNFR2 can be a common target for these two diseases.

## Materials and methods

### Animals

Male C57BL6JBomTac mice (WT) were purchased from the DBL (Daejeon, Korea). The TNF receptor (TNFR) knock-out mice, B6.129Tnfrsf1a < tm1Mak > /J (TNFR1 KO) and B6.129S2Tnfrsf1b < tm1Mwm > /J (TNFR2 KO), were purchased from The Jackson Laboratory (Bar Harbor, ME, US). The mice were housed and bred under specific pathogen-free conditions at the Laboratory Animal Research Center of the Chungbuk National University, Korea (CBNUA-1215-18-01). The mice were maintained in a room with a constant temperature of 22 ± 1 °C, relative humidity of 55 ± 10%, and under a 12/12 h light/dark cycle. Standard rodent chow (Samyang, Gapyeong, Korea) and purified tap water were available ad libitum. The experiments were performed at least 1 week after their arrival in individual home cages.

### Xenograft animal model

A549 cells were injected subcutaneously (1 × 10^7^ tumor cells/100 μl phosphate-buffered saline (PBS)) with a 27-gauge needle into the right lower flank of WT, TNFR1 KO, and TNFR2 KO mice. The weight and tumor volume of the animals were monitored twice per week. The tumor volumes were measured with Vernier calipers and calculated by the following formula: (A × B^2^)/2, where A is the larger and B is the smaller of the two dimensions. At the end of the experiment, the animals were sacrificed with cervical dislocation. The tumors were separated from the surrounding muscles and dermis, excised, and weighed.

### Behavioral test

#### Overview

A549 cells were injected subcutaneously into right lower flank of WT, TNFR1 KO, and TNFR2 KO male mice at 8 week-old. Behavioral tests began at 28 days after the viral vector injection. All tests were performed during 9 am to 6 pm. 6 groups of mice (WT; n = 6, WT + A549; n = 10, TNFR2 KO; n = 7, TNFR2 KO + A549; n = 9, TNFR1 KO; n = 7, and TNFR1 KO + A549; n = 9), was tested for open field, social interaction test, elevated plus maze, T-maze, Y-maze, pre-pulse inhibition (PPI) of acoustic startle, and forced swim tests. This order was determined by the degree of stress and each behavioral test run 48 h interval (Paylor et al. 2006).

#### Open field test (OFT)

Locomotor activity was tested in a 61 × 61 cm open field arena. Mice were independently placed in one corner of the arena and horizontal movements were recorded for 30 min with SMART-LD program (Panlab, Barcelona, Spain). The open field chamber was cleaned between trials with 70% v/v alcohol solution.

#### Social interaction test (SIT)

Before the SIT was conducted, all experimental mice were placed in the laboratory room for acclimation for at least an hour. Each test mouse (from 6 groups) was carefully placed in the center of the social field twice, either with an empty transparent perforated enclosure (first 150 s trial) or with a completely novel C57 mouse contained in the enclosure (150 s trial). Between the two trials, the test mice were placed back in their own cages for a 30 s rest period. The exploratory activity of the test mice in the two trials was recorded and analyzed by a video-tracking with SMART-LD program (Panlab, Barcelona, Spain). For each test mouse, the social interaction (SI) ratio was calculated as (interaction time, C57 present)/(interaction time, C57 absent) × 100%, and the corner ratio was calculated as (corner time, C57 present)/(corner time, C57 absent) × 100%.

#### Elevated plus maze (EPM)

A Plexiglas plus-shaped maze containing two dark enclosed arms and two open arms elevated 50 cm above ground was used to explore basal anxiety-related behaviors. The arms consisted in 30 × 5 cm with a 5 × 5 cm center arena, and the walls of the closed arms were of 20 cm high. Trials started by placing mice in the center of the maze. Mice were video tracked for 5 min and then returned to their home cage. The total time mice spent in the closed/opened arms and center of the maze were scored by SMART-LD program (Panlab). The plus maze was cleaned between trials with 70% v/v alcohol solution.

#### T-maze (TM)

Spontaneous alternation was assessed in an opaque plastic, enclosed T maze. The maze was fitted with plastic sliding doors at the entrance to each goal arm and 23 cm from the beginning of the start arm. The maze was also fitted with a removable, opaque, plastic central partition (15 cm high, 10 cm long) that extended from the top wall of the T maze into the start arm. At the beginning of the test, all the doors were raised, and the central partition was in place. The mouse was placed in the start arm facing the end wall and allowed to enter to any goal arm of its preference. A choice was defined as a mouse placing all four paws and tail inside an arm. Once a choice was made, the door of the goal arm was gently lowered to confine the mouse to the chosen arm during 30 s, and simultaneously removing the central partition device. Then, the mouse was placed back in the start arm and allowed to freely choice of either goal arm. Whether or not the mouse alternated arms were recorded (i.e., alternated or not alternated). There was a delay of 15 s between the sample run and the choice run. Each mouse was tested three times with 90 min of delay between trials.

#### Y-maze (YM)

Spatial novelty preference was assayed by using the Y-maze apparatus, which consisted in three closed arms (30 cm long, 9 cm wide, 10 cm center, and 13 cm of wall high/each) made with opaque blue plastic. Each trial consisted of 2 phases, a 5 min exploration phase and 2 min test phase, separated by a 1 min delay. During the exploration phase mice could explore the Start arm and the other arm, but access to the remaining arm of the Y-maze (the Novel arm) was blocked with a wall of opaque plastic. During the test phase mice were allowed to freely explore all three of the mazes. Timing of test phase was started when the mouse left the start arm by placing all four paws outside the arm. The main outcome measure was the time spent in each arm. Allocation of arms (Start, Other and Novel) to specific location and to the arms of the Y-maze was counterbalanced within each experimental group. The Y-maze was cleaned between trials with 70% v/v alcohol solution. Percentage of novelty arm preference was quantified by calculating the proportion of time in novel arm out of total time spent in all three arms during the test phase (120 s).

#### Pre-pulse inhibition of the startle response (PPI)

The Pan-Lab Startle Response System (Panlab) was used to measure the pre-pulse inhibition of the startle response (PPI). Mice were placed in a clear Plexiglas holding cylinder and, after 5 min acclimation, presented with random trials of startle (40 ms at an intensity of 120 dB), prepulse + startle (20 ms of 68, 71, 77, and 82 dB noise followed, 100 ms later, by 40 ms at an intensity of 120 dB) and nonstartling-stimulus (NSS). A 65 dB background sound was presented throughout the session. Inter-trial intervals averaged 15 s (range: 5–20 s). Percentage of PPI was calculated as [(startle response-NSS)-(prepulse + startle response-NSS)/(startle response-NSS)] × 100.

#### Forced swimming test (FST)

The cylindrical tanks (30 cm height × 20 cm diameters) required for the mouse forced swim test (FST) in our laboratory are constructed of transparent Plexiglas, as this material is able to withstand the frequent movement of the tanks and accidents better than glass. The water level is 15 cm from the bottom and should be marked on the tank to ensure that the volume of water is consistent across mice. Swimming movements were recorded for 6 min by SMART-LD program (Panlab). The data was analyzed with only the last four minutes.

#### Hematoxylin and eosin (H&E) staining and immunohistochemistry (IHC)

After the behavioral tests, mice were perfused with phosphate-buffered saline (PBS, pH 7.4) under inhaled CO2 anesthetization. The tumor tissues and brains were immediately removed from the mice and fixed in 4% paraformaldehyde for 48 h at 4 °C and transferred to 30% sucrose solutions for H&E and IHC staining. The mouse tissues frozen sections were blocked with 1% bovine serum albumin (BSA) diluted in PBS for 1 h at room temperature (RT); the sections were then blotted and incubated with rabbit recombinant antibody for BDNF (abcam, #ab108319) or rabbit polyclonal antibody for TrkB (abcam, #ab18987) at the appropriate dilution (1:40 dilution) in blocking serum for overnight at 4 °C. The slides were washed in PBS, followed by the anti-rabbit IgG-horseradish peroxidase (HRP) (1:500; Cell Signaling Technology, MA, USA #7074S) secondary antibody. The slides were washed, the peroxidase reaction was developed with diaminobenzidine and peroxide, and the slides were mounted in Aqua-Mount and evaluated on a light microscopy (Microscope Axio Imager.A2, Carl Zeiss, Oberkochen, Germany). A negative control was performed by omitting the primary antibody.

### Western blot analysis

The total proteins were extracted from the mice and A549 cells by PRO-PREP™ Protein Extraction Solution (iNtRON Biotechnology, Inc., Seongnam, Korea). Protein was extract by PRO-PREP™ Protein Extraction Solution (iNtRON Biotechnology, Inc., Seongnam, Korea). An equal amount of total protein (20 μg) was resolved on 8–15% sodium dodecyl sulfate polyacrylamide gel and then transferred to a nitrocellulose membrane (Hybond ECL; Amersham Pharmacia Biotech, Piscataway, NJ, USA). The membranes were blocked for 1 h in 2.5% skim milk solution and incubated overnight at 4 °C with specific antibodies. To detect target proteins, specific antibodies against BDNF (abcam, #ab108319), TrkB (abcam, #ab18987), and β-actin (Santa Cruz Biotechnology Inc.) were used (1:1000), overnight incubation at 4 °C. The blots were then incubated with the corresponding conjugated goat anti-rabbit or goat anti-mouse IgG-horseradish peroxidase (HRP) (1:5000; Santa Cruz Biotechnology Inc.) secondary antibodies, 90 min incubation at room temperature. Immunoreactive proteins were detected using enhanced chemiluminescence (ECL) Western blotting detection system (FUSION SOLO S; Vilber Lourmat, Paris, France). The relative density of the protein bands was measured by ImageJ (Wayne Rasband, National Institutes of Health, Bethesda, MD).

### Cell culture and transfection

A549 cells were obtained from the American Type Culture Collection (Manassas, VA). A549 cells were cultured in RPMI 1640 medium supplemented with 10% heat‐inactivated fetal bovine serum (FBS), 100 μg/ml penicillin, and 100 μg/ml streptomycin. Cell cultures were maintained in an incubator with a humidified atmosphere of 5% CO2 at 37 °C. For transfection, A549 cells were transiently transfected with TNFRSF1B siRNA, using RNAiMAX (for siRNA) reagent in Opti-MEM, according to the manufacturer’s specifications. TNFRSF1B siRNA was purchased from Origene (Rockville, MA, USA). Cells were incubated with BDNF (100 ng/ml) for the indicated time points in figure legends.

### Fluorescence microscopy

The fixed cells were exposed to the following primary antibodies: BDNF (1:100, Santa Cruz Biotechnology Inc. Santa Cruz, CA, USA), at room temperature for 2 h. After incubation, the cells were washed twice with ice-cold PBS and incubated with an anti-mouse secondary antibody conjugated to Alexa Fluor 568 nm (Invitrogen-Molecular Probes, Carlsbad, CA) at room temperature for 1 h. Counter stain with TUNEL assay kit (DeadEnd™ Fluorometric TUNEL System, Promega, WI, USA). Immunofluorescence images were acquired using an K1-Fluo laser scanning confocal microscope (Nanoscope Systems, Daejeon, Korea).

### Culture of primary neurons

The 3 days WT mice were anesthetized by CO_2_. Cortical tissue was isolated under sterile conditions, rinsed in Hank's Buffered Saline Solution (HBSS), and minced into small pieces. The minced tissue was then treated with 1 ml trypsin for 15 min at 37 °C. The trypsin/HBSS supernatant was removed and replaced with 5 ml of prewarmed plating medium (DMEM/F12 + 10% fetal calf serum + Antibiotic–antimycotic 1×), after which the tissue was immediately triturated. The dispersed cells were counted and adjusted to of 2.0 × 10^5^ cells/ml. This cell solution was added to L-polyornithine-coated dish. Cells were allowed to settle for 15 min, washed once, and replaced with prewarmed plating medium. After plating, cells were incubated at 37 °C in 5% CO2 and incubated for 11 days without media replacement. On day 11, the plating media was replaced with the same volume of feeding medium (B27 neurobasal media + Antibiotic–antimycotic 1x + Glutamine 2 mM was added to the media on the day of use). The experiments (transfection and Western blot) were conducted on day 14.

### Xenograft and BDNF injection animal model

A549 cells were injected subcutaneously into right lower flank of WT, TNFR1 KO, and TNFR2 KO mice at 8 week-old. Mice also received intravenous (i.v.) injections of BDNF (160 μg/kg), every 4 days including the first day of xenograft, for a total of 8 injections. Behavioral tests began at 28 days after the viral vector injection. All tests were performed during 9 am to 6 pm. 6 groups of mice (WT; n = 8, WT + A549; n = 8, WT + BDNF + A549; n = 8, TNFR2 KO; n = 8, TNFR2 KO + A549; n = 8, TNFR2 KO + BDNF + A549; n = 8, TNFR1 KO; n = 8, TNFR1 KO + A549; n = 8, TNFR1 KO + BDNF + A549; n = 8,), was tested for open field, social interaction test, elevated plus maze, T-maze, Y-maze, pre-pulse inhibition (PPI) of acoustic startle, and forced swim tests. This order was determined by the degree of stress and each behavioral test run 48 h interval (Paylor et al. 2006). At the end of the study, the mice were euthanized via CO2 inhalation and perfused with PBS immediately followed by 4% formalin fixative solution.

### BDNF assay

Samples for analysis were collected after the behavioral tests. Serum and tissue levels of mouse BDNF was measured by enzyme-linked immunosorbent assay (ELISA) kits provided by R&D systems (Minneapolis, MN, USA) according to the manufacturer’s protocol. Assay samples were collected after end of behavior tests.

### Web‐based analysis

The disease-disease and gene-disease association data were analyzed by DisGeNET (https://www.disgenet.org/) and open target platform (https://platform.opentargets.org/). The gene–gene interaction data was analyzed by genemania (https://genemania.org/).

### Statistical analysis

Statistical analyses were performed using GraphPad Prism v8.4.2 and R. All data was examined for normal distribution with D’Agostino & Pearson normality test. If data set exhibited normal distribution, we performed parametric tests. To analyze data measured over two or more different conditions, we used two-way ANOVA for repeated measures and Holm-Sidak post-hoc analysis. If effect sizes of data are less than 0.8 or the sample size is less than 10 per group, we performed permutation tests for group comparisons. Data are represented as the mean ± S.E.M., and significance set at p < 0.05.

## Results

### SCZ association with lung cancer through TNFR2

**Fig. 1:**
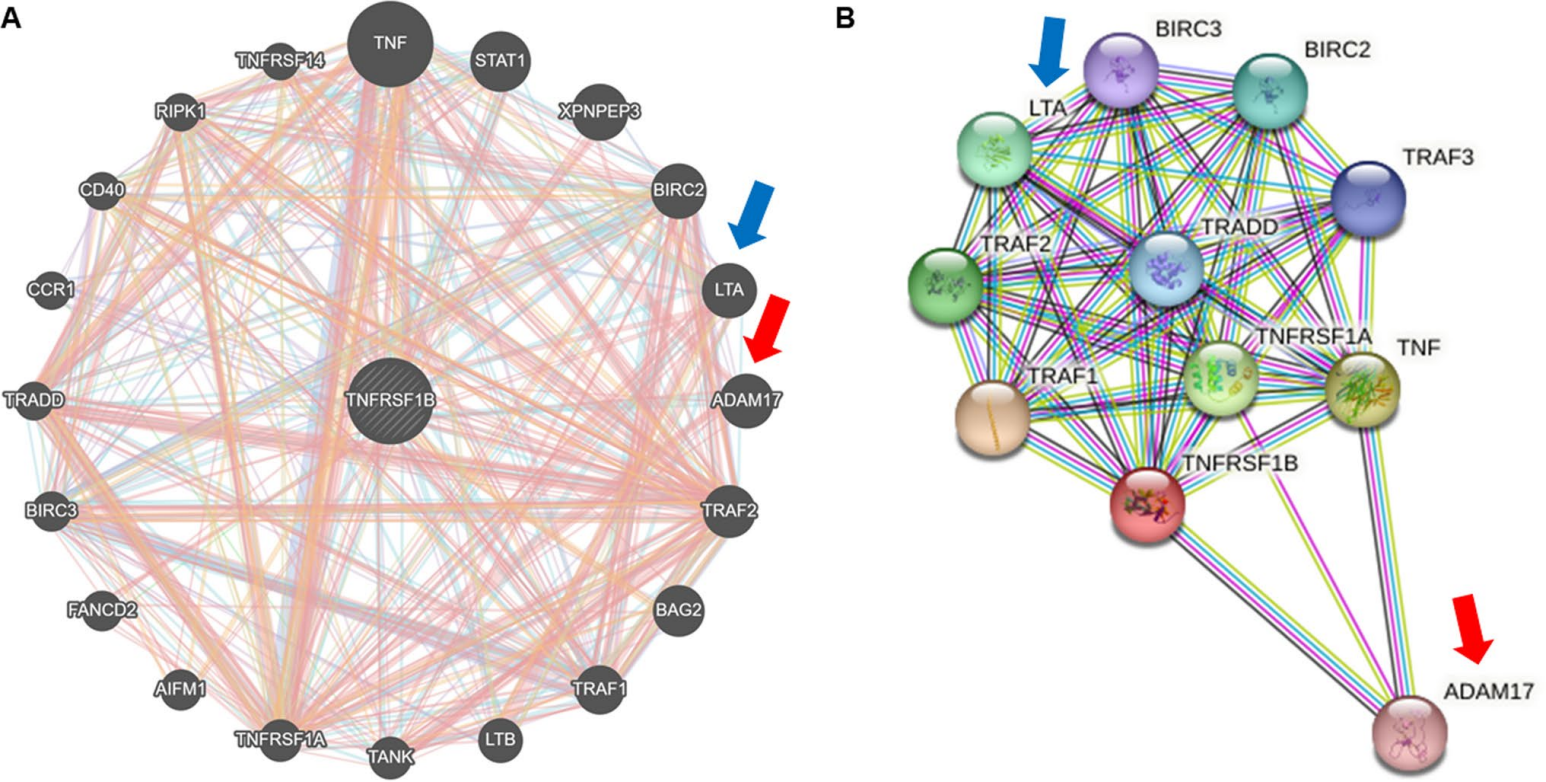
Predicted functional associations between genes and proteins with TNFR2 (TNFRSF1B). Gene-association network analysis by Genemania (https://genemania.org/) (**A**), and protein-association network analysis by String database (https://string-db.org/) (**B**) with TNFR2 (TNFRSF1B). In the data of (**A**), a larger relationship with TNFR2 was shown in clockwise order. Red and blue arrows are point to common targets in (**A**) and (**B**). These two data were analyzed in *homo sapiens*

To determine the association between schizophrenia and tumor, we analyzed the DisGeNet database (https://disgenet.org/) (Piñero et al. 2016). The results showed that schizophrenia is associated with several neuronal diseases. However, various cancers such as primary malignant neoplasms, malignant neoplasms, malignant neoplasms of the prostate, and breast, lung carcinomas, and malignant neoplasms of the lungs were also associated with SCZ (Table 1). Thirty-six diseases showed more than 0.18 Jaccard index based on shared genes (JIg) values which highly indicates the association with SCZ. Among them, 18 diseases were cancers, and most of them had more than 1000 shared genes. Next, we analyzed the significance of TNFR2 in the development of disease using DisGeNet, and SCZ was ranked 7th out of 417 diseases with a gene-disease association score of 0.33 (Table 2). Cancer was ranked 13th out of 434 diseases by the open targets platform (https://platform.opentargets.org/), a notable difference is that this platform provides additional target-disease association through approved drugs and clinical candidates, RNA expression, and biological pathways disrupted by genetic mutations information that is not available in DisGeNET (Carvalho-Silva et al. 2019), with an overall association score of 0.11 (Table 3). To find the TNFR2-associated target gene, we used GeneMANIA (http://genemania.org) for analyzing the gene–gene function association network (Fig. 1A) and used STRING (http://genemania.org) for the protein–protein function association network (Fig. 1B). From these two data, commonly lymphotoxin alpha (LTA) and a disintegrin and metalloprotease 17 (ADAM17) were identified to be most associated to TNFR2. LTA is the genetic polymorphism variant in SCZ (Arab and Elhawary 2015). ADAM17 is an emerging therapeutic target for lung cancer and its expression has been reported to be associated with SCZ (Hoseth et al. 2017; Saad et al. 2019). These results obtained by data analysis indicate that SCZ is significantly associated with lung cancer, and TNFR could be a common target for the comorbidity of the two diseases.

**Table 1:** Cluster of disease-disease associations with schizophrenia

Associated disease	Number of shared genes	JIg	Associated disease	Number of shared genes	JIg
Alzheimer’s disease	1261	0.2518	Obesity	912	0.1908
Bipolar disorder	803	0.2469	Malignant neoplasm of breast^a^	1572	0.1908
Depressive disorder	874	0.2351	Pain	709	0.1907
Mental depression	787	0.2209	Malignant neoplasms^a^	1839	0.1905
Depressed mood	766	0.2147	Prostate carcinoma^a^	1140	0.1863
Parkinson disease	867	0.2123	Glioblastoma multiforme^a^	950	0.1856
Diabetes mellitus, non-insulin-dependent	1028	0.2065	Colorectal carcinoma^a^	1303	0.185
Diabetes mellitus	971	0.2064	Melanoma^a^	926	0.184
Impaired cognition	748	0.1993	Carcinoma of lung^a^	1080	0.1839
Major depressive disorder	681	0.1987	Malignant neoplasm of prostate^a^	1145	0.1838
Neuroblastoma^a^	891	0.1984	Glioblastoma^a^	939	0.1838
Diabetes	862	0.1973	Malignant neoplasm of lung^a^	1092	0.1834
Central neuroblastoma^a^	867	0.196	Neurodegenerative disorders	676	0.1822
Childhood neuroblastoma^a^	867	0.1959	Neoplasms^a^	2005	0.1818
Breast carcinoma^a^	1574	0.1949	Hypertensive disease	798	0.1815
Rheumatoid arthritis	903	0.1925	Neoplasm metastasis^a^	1422	0.1815
Seizures	809	0.1919	Primary malignant neoplasm of lung^a^	1032	0.18
Primary malignant neoplasm^a^	1783	0.1915	Multiple sclerosis	712	0.1798

Disease-disease associations listed by Jaccard index based on shared genes with schizophrenia were selected from the DisGeNet database

^a^Diseases related to cancer, JIg: Jaccard index based on shared genes

**Table 2:** Diseases or phenotypes associated with TNFR2 (DisGeNET)

Associated disease	Gene-disease association score
Mycosis fungoides	0.51
Sezary syndrome	0.51
Diabetes mellitus, non-insulin-dependent	0.39
Obesity	0.36
Depressive disorder	0.34
Mental depression	0.34
Schizophrenia	0.33
Paranoid schizophrenia	0.33
Autistic disorder	0.31
Pneumonia	0.31
Pneumonitis	0.31
Liver cirrhosis	0.3
Non-alcoholic fatty liver disease	0.3
Hypersensitivity	0.3
Nonalcoholic steatohepatitis	0.3
Brain ischemia	0.3
Cerebral ischemia	0.3
Experimental lung inflammation	0.3

Full data of 417 diseases information is in supplementary Table 1

**Table 3:** Diseases or phenotypes associated with TNFR2 (open targets platform)

Associated disease	Overall association score
Eosinophil count	0.426510085
Eosinophil percentage of leukocytes	0.379266023
Tumor necrosis factor receptor II measurement	0.290225537
Blood protein measurement	0.288841012
Autoimmune disease	0.286160938
Hypothyroidism	0.262928681
Glioblastoma multiforme	0.185529087
Lymphocyte count	0.165588588
Gastric adenocarcinoma	0.150211365
Asthma	0.118581956
Neoplasm	0.116304242
Chronic kidney disease	0.106942289
Cancer	0.106483044
Experimental autoimmune encephalomyelitis	0.097312998
Rheumatoid arthritis	0.095299851
Infect	0.093675145
Infection	0.093675145
Type 2 diabetes mellitus	0.092731111

Full data of 434 diseases information is in supplementary Table 2

### Deletion of TNFR2 significantly reduced tumor growth

**Fig. 2:**
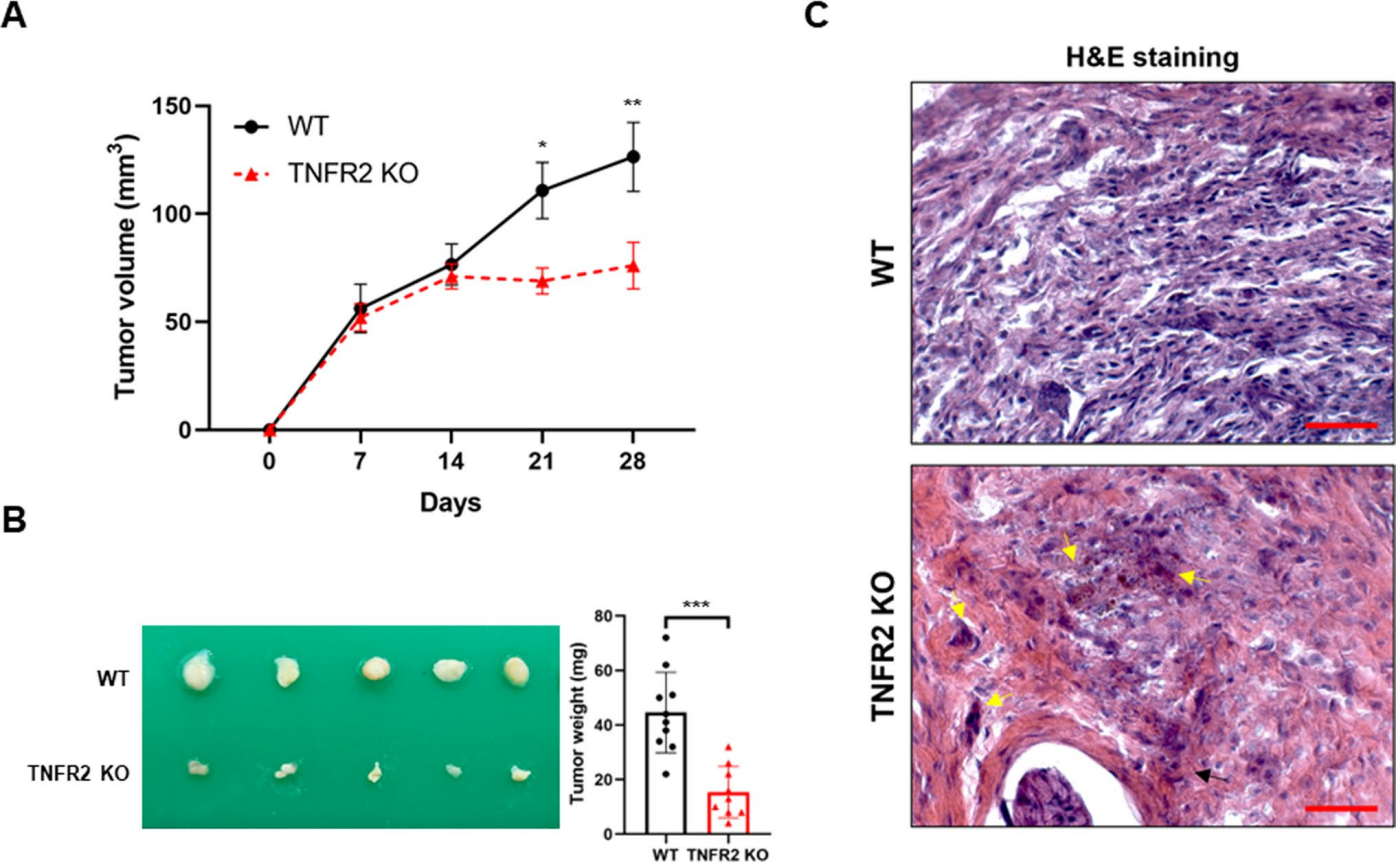
Decreased tumor growth in the TNFR2 KO mice xenografted with A549 lung cancer cells. Cancer cells (A549) were injected into the right lower flank in mice. All data indicate tumors formed on the right lower flank. Tumor volumes (**A**) and weights (**B**). Points and columns, means of animals (WT + A549; n = 10, TNFR2 KO + A549; n = 9). Values are means ± SEM. *p < 0.05, **p < 0.005, ***p < 0.001 compared with the WT mice. Tumor sections were analyzed by H&E staining (**C**). Yellow arrows indicate apoptotic cells. H&E staining were repeated from three independent experiments. Scale bars represent 100 μm

We previously found that TNFR2 promotes SCZ-like behavior in an in vivo model (Yeo et al. 2022). To verify the roles of TNFR2 in lung tumor growth and SCZ, we used a xenograft model in TNFR2 KO transgenic mice. Tumor development was monitored for 28 days. The tumor volume (day 21: WT, 110.83 ± 12.96; TNFR2 KO, 68.96 ± 5.99 mm^3^, day 28: WT, 126.42 ± 15.93; TNFR2 KO, 76.08 ± 10.77 mm^3^, Fig. 2A) and weight (WT, 44.60 ± 14.77 mg; TNFR2 KO, 15.44 ± 9.40 mg, Fig. 2B) in TNFR2 KO mice were significantly lowered compared with the WT mice. Hematoxylin and eosin (H&E) staining revealed that the WT tumor tissues had large nuclei and tight spaces, and were present in mitotic cells, characteristics of cancer cells. However, in TNFR2 KO mice tumor tissues, the number of mitotic cells was lower than in WT mice tumor tissues, and the number of dead cells was higher (Fig. 2C). We also found that the TNFR1 KO suppressed xenograft lung tumor growth and volume, but tumor volumes were not significantly reduced compared to WT (Fig. S1A and B). In TNFR1 KO mice xenografted with A549 lung cancer cells tumor tissue, tumor weights were significantly lower than WT, but not much than TNFR2 KO mice xenografted with A549 lung cancer cells. These results suggest that the deletion of TNFR2 greatly interferes with tumor growth.

### Deletion of TNFR2 significantly reduced SCZ-like behaviors

**Fig. 3:**
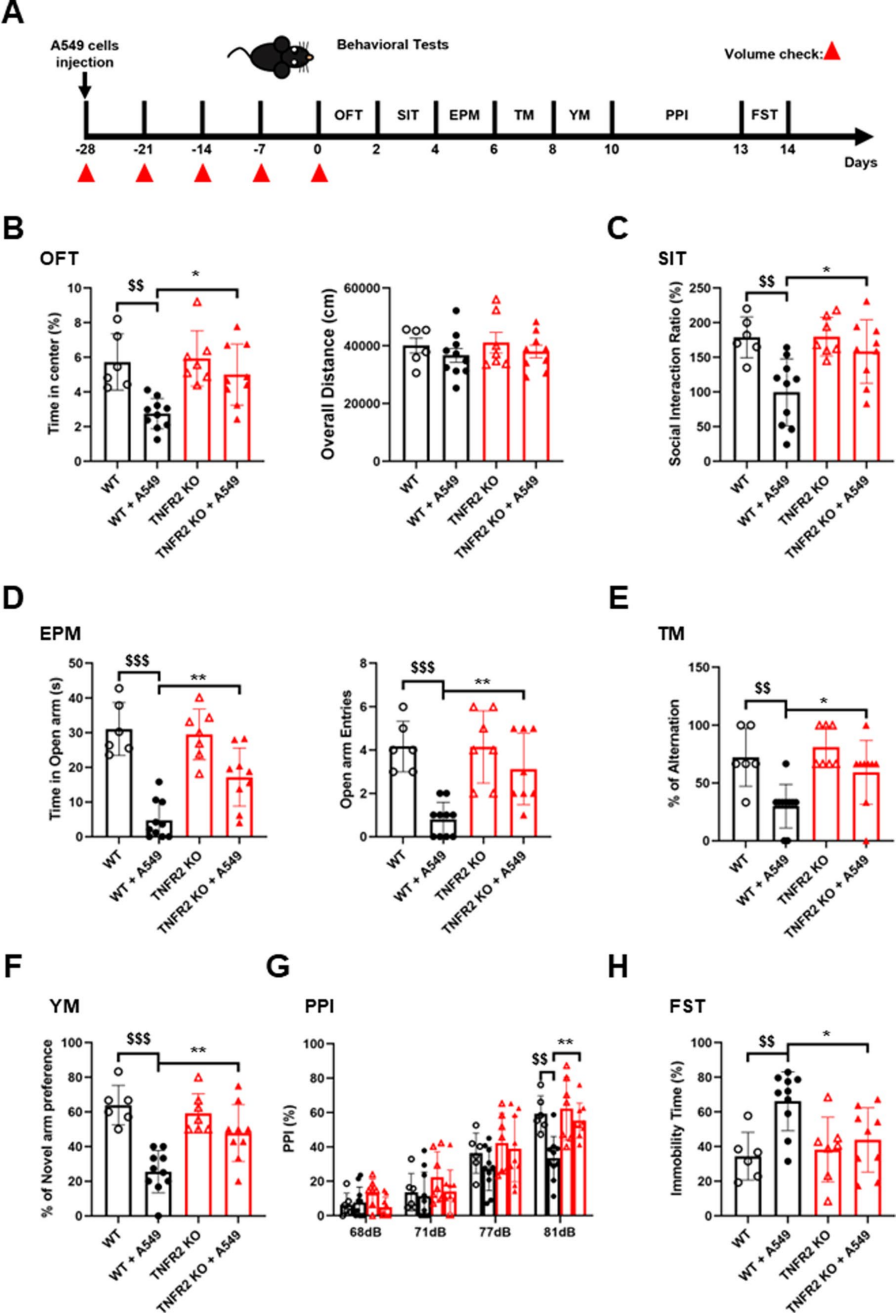
No significant effect on SCZ-like behavior by xenograft in the TNFR2 KO mice xenografted with A549 lung cancer cells. Timeline of behavioral tests for mice xenografted with A549 lung cancer cells (**A**). Locomotor activity and anxiety measured as time in central zone (%) and overall distance (cm) in open field test (OFT) (**B**). Social anxiety disorder was measured as time in interaction zone (%) in social interaction test (SIT) (**C**). Basal anxiety assessed as time (in sec) and entries in open arms in the elevated plus maze (EPM) (**D**). Cognitive impairment was measured by T-maze (TM) (**E**) and Y-maze (YM) (**F**). Sensorimotor gating was measured by pre-pulse inhibition test (PPI) (**G**). Depression immobility time (in sec) in forced swimming test (FST) (**H**). Data are represented as means ± SEM (WT; n = 6, WT + A549; n = 10, TNFR2 KO; n = 7, TNFR2 KO + A549; n = 9). ^$$^p < 0.005, ^$$$^p < 0.001 compared with the WT + A549 mice. *p < 0.05, **p < 0.005 compared with the TNFR2 KO + A549 mice

To identify the relationship between tumor growth and SCZ development, behavioral tests were performed in TNFR2 KO mice xenografted A549 lung cancer cells for 28 days (Fig. 3A). Since SCZ is accompanied by complex symptoms, various behavioral experiments were performed, OFT (Fig. 3B) for locomotor and anxiety, SIT (Fig. 3C) for social withdrawal, EPM (Fig. 3D) for anxiety, T-maze (Fig. 3E) and Y-maze (Fig. 3F) for cognitive symptom, PPI (Fig. 3G) for sensorimotor gating, and FST (Fig. 3H) for depression. All tests were performed between 9 am to 6 pm and there was a 48-h rest period between each test. Between WT and TNFR2 KO mice without cancer cells xenograft, there was no significant difference in these behavioral tests. Based on the OFT results, it was observed that tumor growth led to an increase in negative symptoms (reduced time spent in the center) in WT mice, while this effect was not observed inTNFR2 KO mice. However, positive symptom (total traveled distance) in OFT results indicated that this xenografted mice did not cause a sickness phenotype. Other negative symptoms in SIT, EPM, and FST were significantly different measured between WT mice WT mice xenografted with A549 lung cancer cells, while no differences were observed in TNFR2 KO mice xenografted with A549 lung cancer cells. Additionally, cognitive symptoms in TM, YM, and PPI were significantly decreased in WT mice xenografted with A549 lung cancer cells, while no changes were observed inTNFR2 KO mice xenografted with A549 lung cancer cells. However, in TNFR1 KO mice, a few behavior test results (EPM and PPI) were significantly decreased by tumor growth like in WT mice (Figure S2). These results suggest that the inhibition of SCZ-like behavior (especially negative and cognitive symptoms) in TNFR2 KO mice could be associated with tumor growth.

### Expression of BDNF and TrkB in the tumor tissue and brain PFC of WT and TNFR2 KO mice

**Fig. 4:**
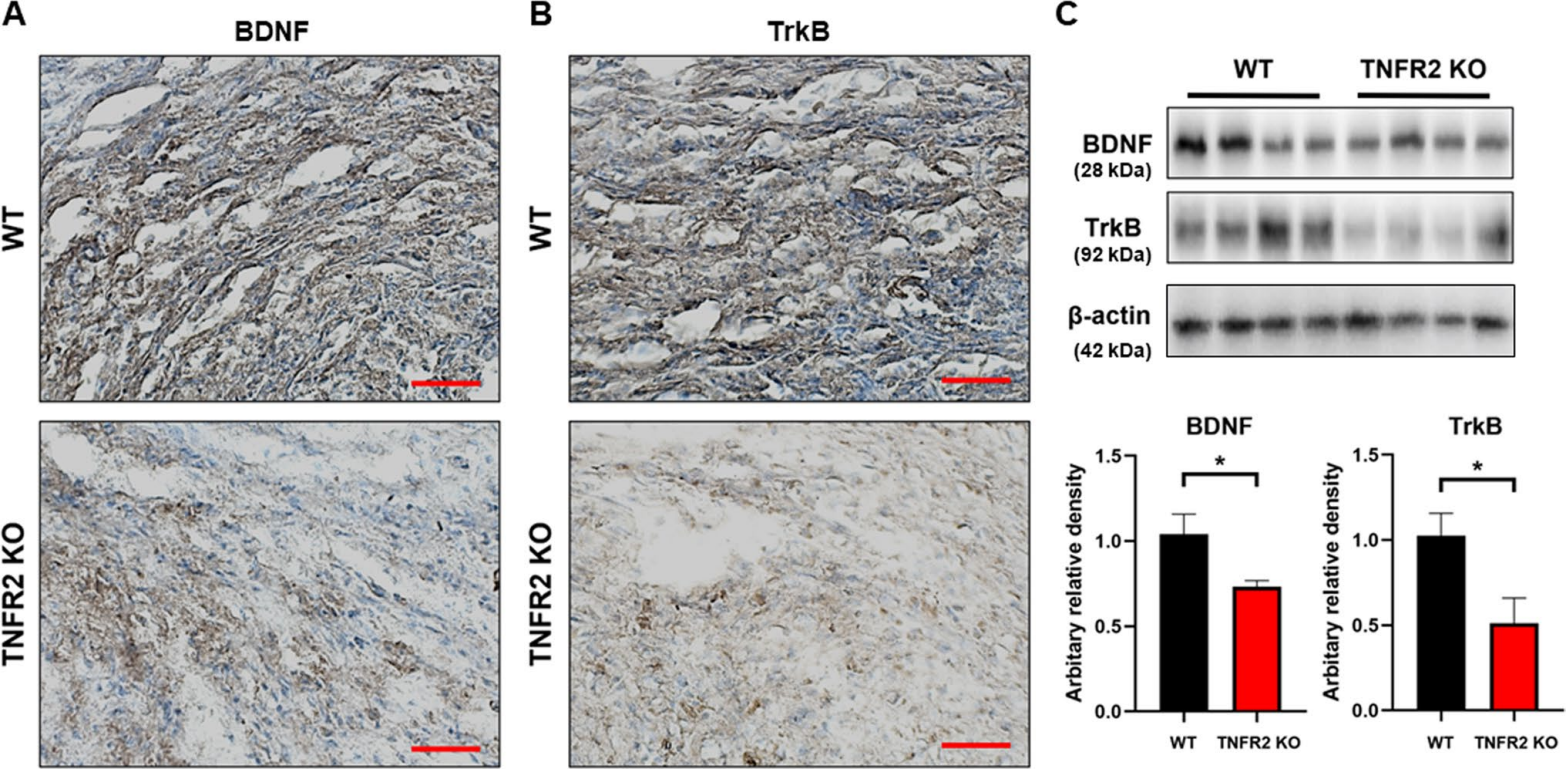
Decreased expression of BDNF and TrkB in the TNFR2 KO mice xenografted with A549 lung cancer cells tumor tissues. Tumor sections were analyzed by immunohistochemistry (BDNF; **A**, TrkB; **B**). All slides were counterstained by hematoxylin. Immunohistochemistry was repeated from three independent experiments. Scale bars represent 100 μm. Tumor extracts were analyzed by Western blotting. Samples (20 μg) were resolved on 12% SDS–PAGE and detected with antibodies against BDNF, TrkB, and β-actin (**C**). β-actin was internal control. The values on the Western blot bands represent the arbitrary density measured by ImageJ. Values are means ± SEM. *p < 0.05 compared with the WT mice

**Fig. 5:**
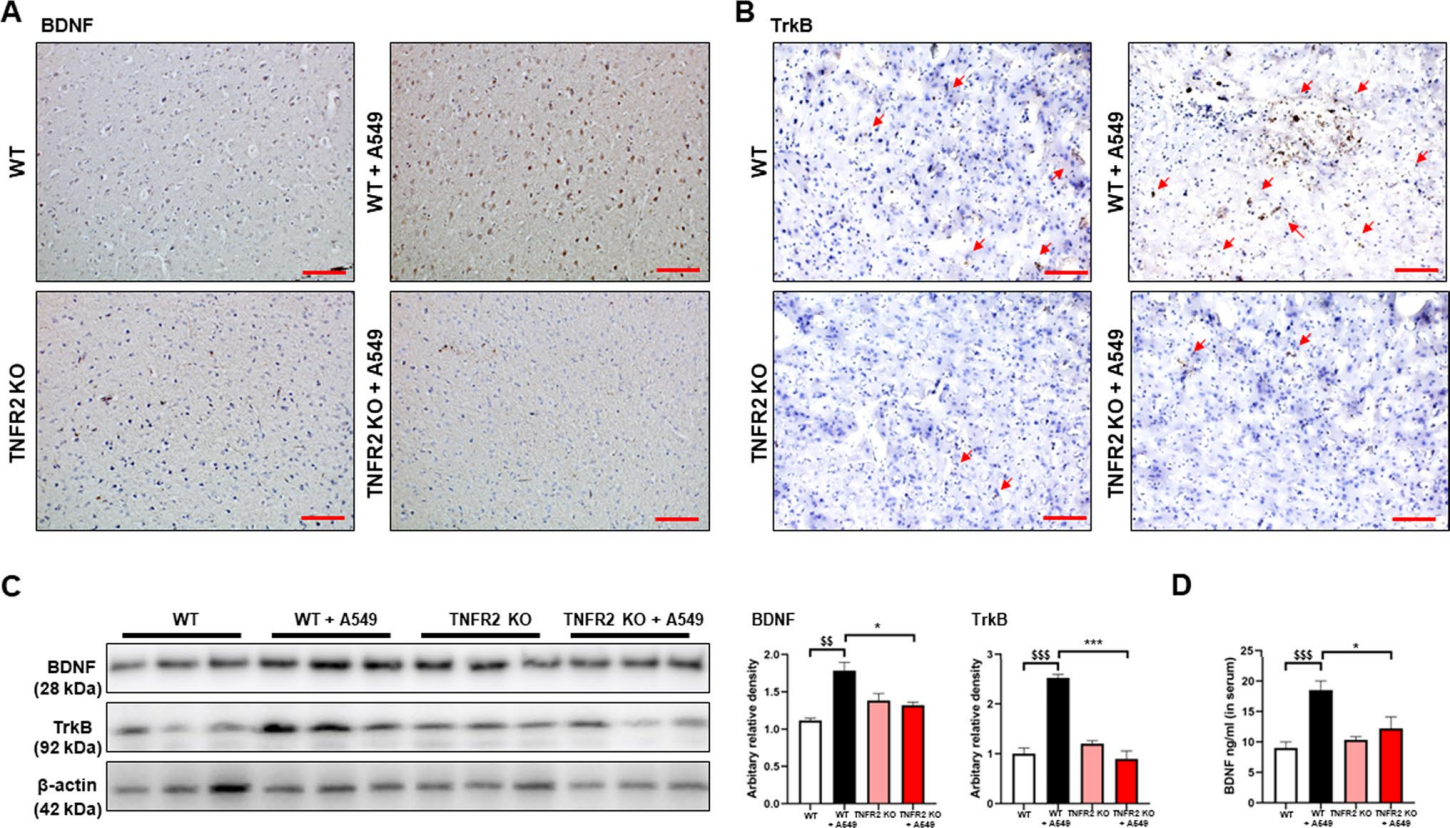
No significant effect on expression level of BDNF and TrkB by xenograft in TNFR2 KO mice brain. Brain sections were analyzed by immunohistochemistry (BDNF; **A**, TrkB; **B**). All slides were counterstained by hematoxylin. Immunohistochemistry was repeated from three independent experiments. Scale bars represent 100 μm. Brain extracts were analyzed by Western blotting. Samples (20 μg) were resolved on 12% SDS–PAGE and detected with antibodies against BDNF, TrkB, and β-actin (**C**). β-actin was internal control. The values on the Western blot bands represent the arbitrary density measured by ImageJ. Values are means ± SEM. *p < 0.05, **p < 0.005, ***p < 0.001 compared with the WT + A549 mice. The protein levels of BDNF were detected by ELISA in mice serum (**D**). $p < 0.05, $$p < 0.005, $$$p < 0.001 compared with the WT + A549 mice

BDNF is a well-known factor that contributes to the development of SCZ and tumor (Chen et al. 2016; Radin and Patel 2017; Di Carlo et al. 2019). It is also noteworthy that the expression of BDNF could be regulated by TNF-α (Zhang et al. 2018). We thus determined the expression of BDNF and TrkB to see whether these signals contribute to TNFR2-induced tumor development. As expected, we found that the TNFR2 KO reduced BDNF and TrkB expression compared with the WT xenograft lung tumor tissues (Fig. 4A and B). Western blotting also showed a decreased level of BDNF and TrkB expression in TNFR2 KO xenograft tumor tissue (Fig. 4C). We next studied the expression of BDNF and TrkB in the mice’s brains. In the previous study, we found that an important area in the brain of SCZ patients was the prefrontal cortex (PFC). Thus, we examined the BDNF and TrkB expression in the PFC of TNFR2 KO and WT mice brains. Immunohistochemical analysis revealed that the BDNF and TrkB reactive cell number between TNFR2 KO and TNFR2 KO mice xenografted with A549 lung cancer cells brains was not significant, whereas significant results were seen in WT and WT A549 lung cancer cells xenografted mouse brains (Fig. 5A and B). The expression of BDNF and TrkB by Western blotting was significantly increased in WT A549 lung cancer cells xenografted mice PFC compared to WT mice PFC, whereas the expression in TNFR2 KO mice xenografted with A549 lung cancer cells PFC was not significant compared to TNFR2 KO mice PFC (Fig. 5C). The level of BDNF in serum was significantly increased in WT A549 xenografted mice compared to WT mice, whereas no significant expression was observed in TNFR2 KO mice xenografted with A549 lung cancer cells compared to TNFR2 KO mice (Fig. 5D). These data indicate that, lung cancer A549 cells xenograft-induced higher levels of BDNF and TrkB expression are important for both tumor growth and SCZ-like behavior and may be closely related to TNFR2 expression.

### Knock down of TNFR2 decreased cell growth, and BDNF and TrkB expression in cultured A549 cells

**Fig. 6:**
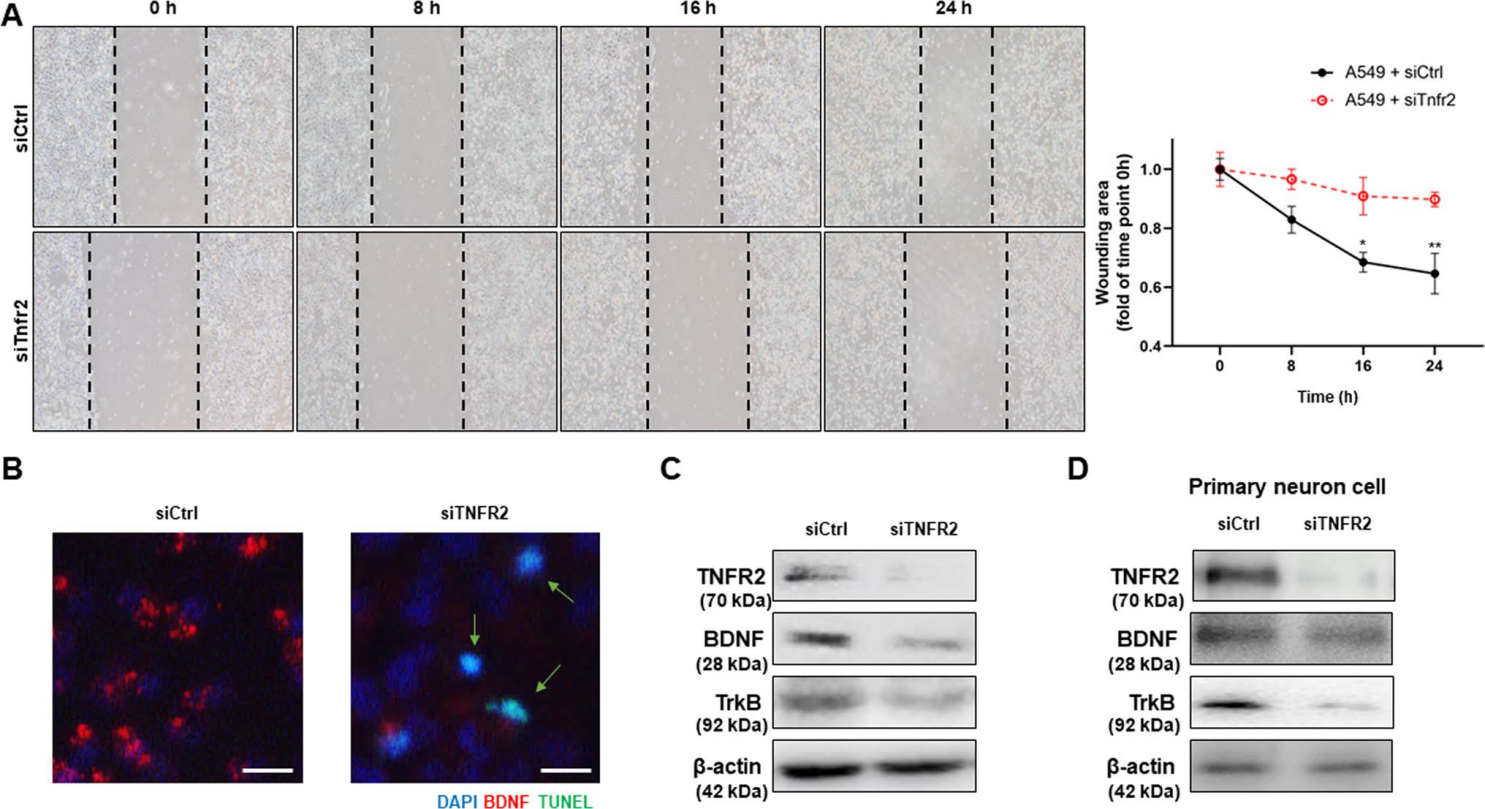
Decreased cell growth and expression of BDNF and TrkB in siTnfr2 transfected A549 cells. Wound healing assay (**A**). Two groups (group 1: siCtrl transfected A549 cells, group 2: siTnfr2 transfected A549 cells) were cultured for 24 h, and the result were quantitatively represented using the curve, which indicated that the group 2 (A549 + siTnfr2) exhibited the lower degree of healing. *p < 0.05, **p < 0.005 compared with the A549 + siCtrl. After 24 h of culture, A549 cells were analyzed by immunofluorescence (BDNF: red) counterstain with TUNEL (green) assay (**B**). Arrows indicate apoptotic cells. Immunofluorescence was repeated from three independent experiments. Scale bars represent 30 μm. Cell extracts (A549 cells: **C**, primary neuron cells: **D**) were analyzed by Western blotting. Samples (20 μg) were resolved on 12% SDS–PAGE and detected with antibodies against BDNF, TrkB, and β-actin. β-actin was internal control

Our in vivo study demonstrated that TNFR2 deficiency decreased tumor growth and SCZ-like behaviors by reducing BDNF and TrkB expression. To further study the effect of TNFR2, we transfected siRNA (siCtrl and siTnfr2) into A549 cells for 24 h. We observed cell growth by wound healing assay. siTnfr2 transfected A549 cells showed reduced healing area, but no reduction was found in siCtrl transfected A549 cells (Fig. 6A). Then we examined BDNF expression and performed a TUNEL assay for assessing cell death based on immunofluorescence at 24 h after siRNA transfection in A549 cells. Decreased BDNF expression and increased cell death were found in siTnfr2 transfected A549 cells (Fig. 6B). The expression of BDNF and TrkB in siTnfr2 transfected A549 cells was significantly decreased compared to siCtrl transfected A549 cells (Fig. 6C). We also examined the expression of BDNF and TrkB in primary neuron culture cells to determine the effect of TNFR2 knockdown. The protein expression of BDNF and TrkB in siTnfr2 transfected primary neuron culture cells was significantly decreased compared to siCtrl transfected primary neuronal cells (Fig. 6D). The in vitro results showed a similar trend as the in vivo results, thereby suggesting that TNFR2 is an important factor in the expression of BDNF and TrkB.

### BDNF injection reduced TNFR2 KO effects on tumor growth and increased expression of BDNF and TrkB

**Fig. 7:**
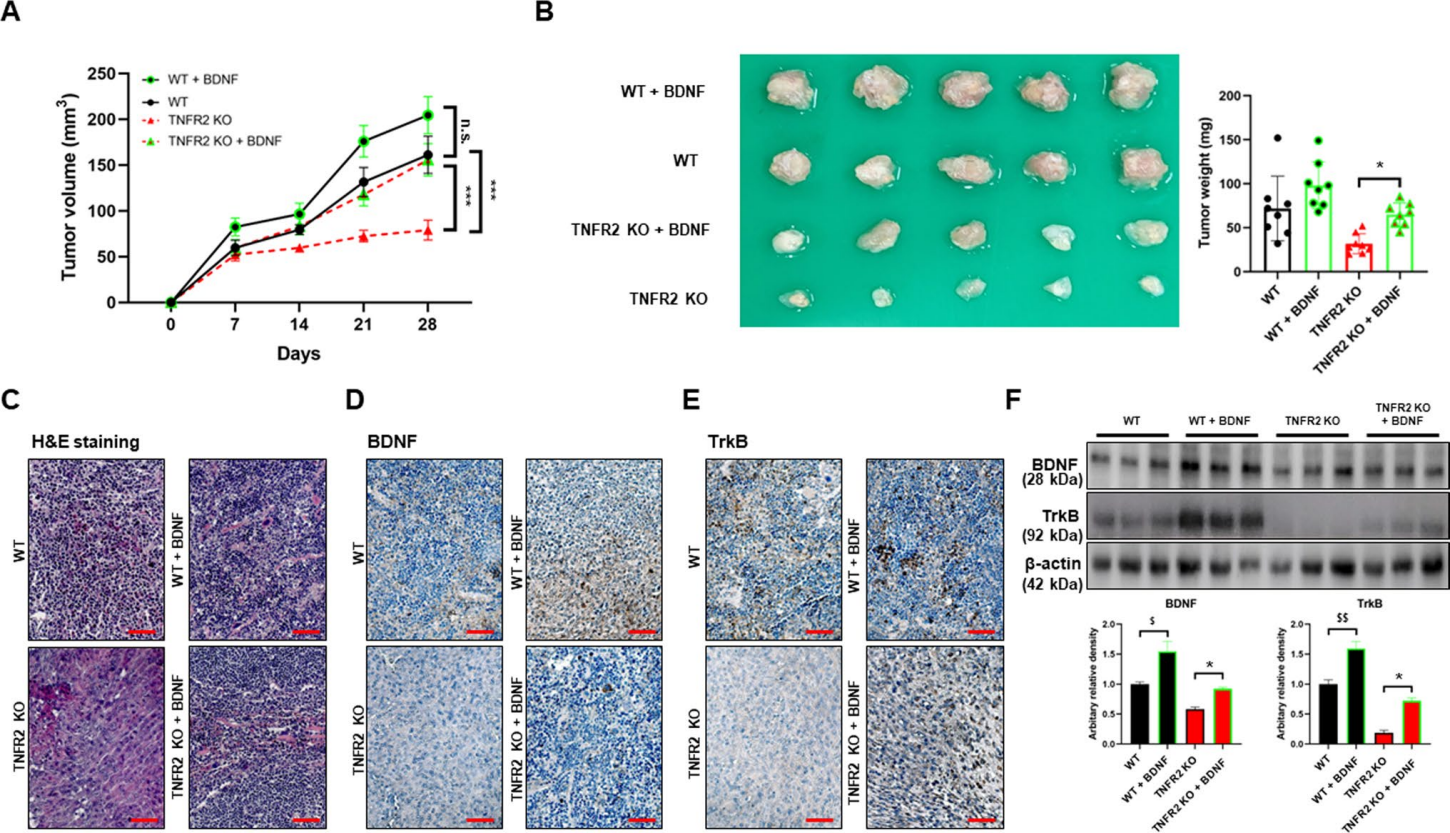
Increased tumor growth and expression of BDNF and TrkB in the BDNF injected TNFR2 KO xenografted mice. Cancer cells (A549) were injected into the right lower flank in mice. All data indicate tumors formed on the right lower flank. Tumor volumes (**A**) and weights (**B**). Points and columns, means of animals (WT; n = 5, WT + BDNF; n = 5, TNFR2 KO; n = 5, TNFR2 KO + BDNF; n = 5). Values are means ± SEM. *p < 0.05, ***p < 0.001 compared with the TNFR2 KO mice. Tumor sections were analyzed by H&E staining (**C**). H&E staining were repeated from three independent experiments. Tumor sections were analyzed by immunohistochemistry (BDNF; **D**, TrkB; **E**). All slides were counterstained by hematoxylin. Immunohistochemistry was repeated from three independent experiments. Scale bars represent 100 μm. Tumor extracts were analyzed by Western blotting. Samples (20 μg) were resolved on 12% SDS–PAGE and detected with antibodies against BDNF, TrkB, and β-actin (**F**). β-actin was internal control. The values on the Western blot bands represent the arbitrary density measured by ImageJ. Values are means ± SEM. *p < 0.05 compared with the TNFR2 KO mice

To verify the roles of BDNF in mediating the effect of TNFR2 KO in lung tumor growth and SCZ, and expression of BDNF and TrkB, we injected BDNF (by intravenous injection, 160 μg/kg every 4 days for 28 days including the first day of xenograft) in our A549 lung cancer cells xenograft mice model. Tumor development was monitored for 28 days. There was a significant difference in tumor growth between the BDNF-injected TNFR2 KO mice xenografted with A549 lung cancer cells and the TNFR2 KO mice xenografted with A549 lung cancer cells. The tumor volume (TNFR2 KO, 79.10 ± 10.86; BDNF injected TNFR2 KO, 155.78 ± 17.62 mm^3^, Fig. 7A) and weight (TNFR2 KO, 31.75 ± 11.27 mg; BDNF injected TNFR2 KO, 65.00 ± 13.22 mg, Fig. 7B) in BDNF injected TNFR2 KO mice xenografted with A549 lung cancer cells were significantly higher compared with the TNFR2 KO mice xenografted with A549 lung cancer cells. Hematoxylin and eosin (H&E) staining revealed that the BDNF-injected TNFR2 KO xenograft tumor tissues had large nuclei and tight spaces, and were present in mitotic cells, characteristics of cancer cells (Fig. 7C). We also found that the expression of BDNF and TrkB was higher in BDNF injected TNFR2 KO A549 lung cancer cells xenograft tumor tissues (Fig. 7D and 7E). The expression level of BDNF and TrkB was also higher in BDNF-injected TNFR2 KO mice xenografted with A549 lung cancer cells tumor tissue (Fig. 7F). These results suggest that exogenous BDNF reduced the inhibitory effect of TNFR2 KO in tumor growth, and the expression of BDNF and TrkB. Thus, these data indicate that TNFR2 is an important mediator in tumor growth as it induces BDNF and TrkB expression.

### BDNF injection abolished TNFR2 KO effect on SCZ-like behavior

**Fig. 8:**
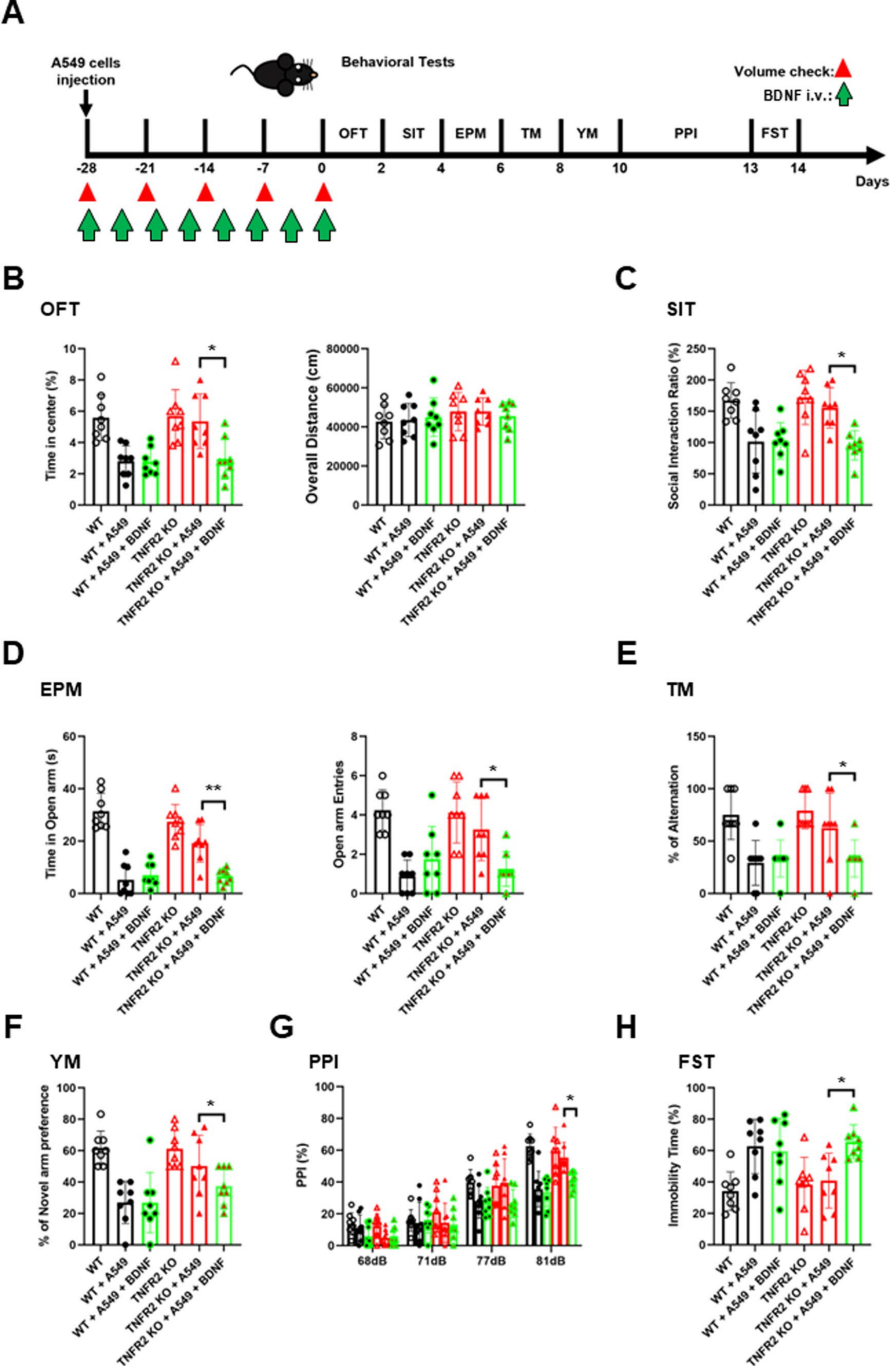
Significant effect on SCZ-like behaviors by BDNF injection in TNFR2 KO xenografted mice. Locomotor activity and anxiety measured as time in central zone (%) in open field test (OFT) (**A**). Social anxiety disorder was measured as time in interaction zone (%) in social interaction test (SIT) (**B**). Basal anxiety assessed as time (in sec) and entries in open arms in the elevated plus maze (EPM) (**C**). Cognitive impairment was measured by T-maze (TM) (**D**) and Y-maze (YM) (**E**). Sensorimotor gating was measured by pre-pulse inhibition test (PPI) (**F**). Depression immobility time (in sec) in forced swimming test (FST) (**G**). Data are represented as means ± SEM (WT; n = 5, WT + A549; n = 5, WT + A549 + BDNF; n = 5, TNFR2 KO; n = 5, TNFR2 KO + A549; n = 5, TNFR2 KO + A549 + BDNF; n = 5). *p < 0.05, **p < 0.005 compared with the TNFR2 KO + A549 mice

We next performed the behavior test in BDNF-injected TNFR2 KO mice xenografted with A549 lung cancer cells. Behavioral experiments were performed according to the stress intensity (Fig. 8A). In previous results (Fig. 3), no SCZ-like behavioral changes were observed in TNFR2 KO mice xenografted with A549 lung cancer cells, but significant SCZ-like behavioral changes were observed when BDNF was co-injected into these mice. Significant decrease in the behavioral tests, OFT (Fig. 8B) for locomotor and anxiety, SIT (Fig. 8C) for social withdrawal, EPM (Fig. 8D) for anxiety, T-maze (Fig. 8E) and Y-maze (Fig. 8F) for cognitive symptom, PPI (Fig. 8G) for sensorimotor gating was observed, but a significant increase was noted in immobility time in FST (Fig. 8H) for depression. As the significant tumor growth by BDNF injection, significant increase of SCZ-like behavior was observed in TNFR2 KO mice xenografted with A549 lung cancer cells. However, BDNF injection did not induce any change in SCZ-like behavior in WT mice xenografted with A549 lung cancer cells.

### Expression of BDNF and TrkB was significantly increased in BDNF-injected TNFR2 KO mice xenografted with A549 lung cancer cells

**Fig. 9:**
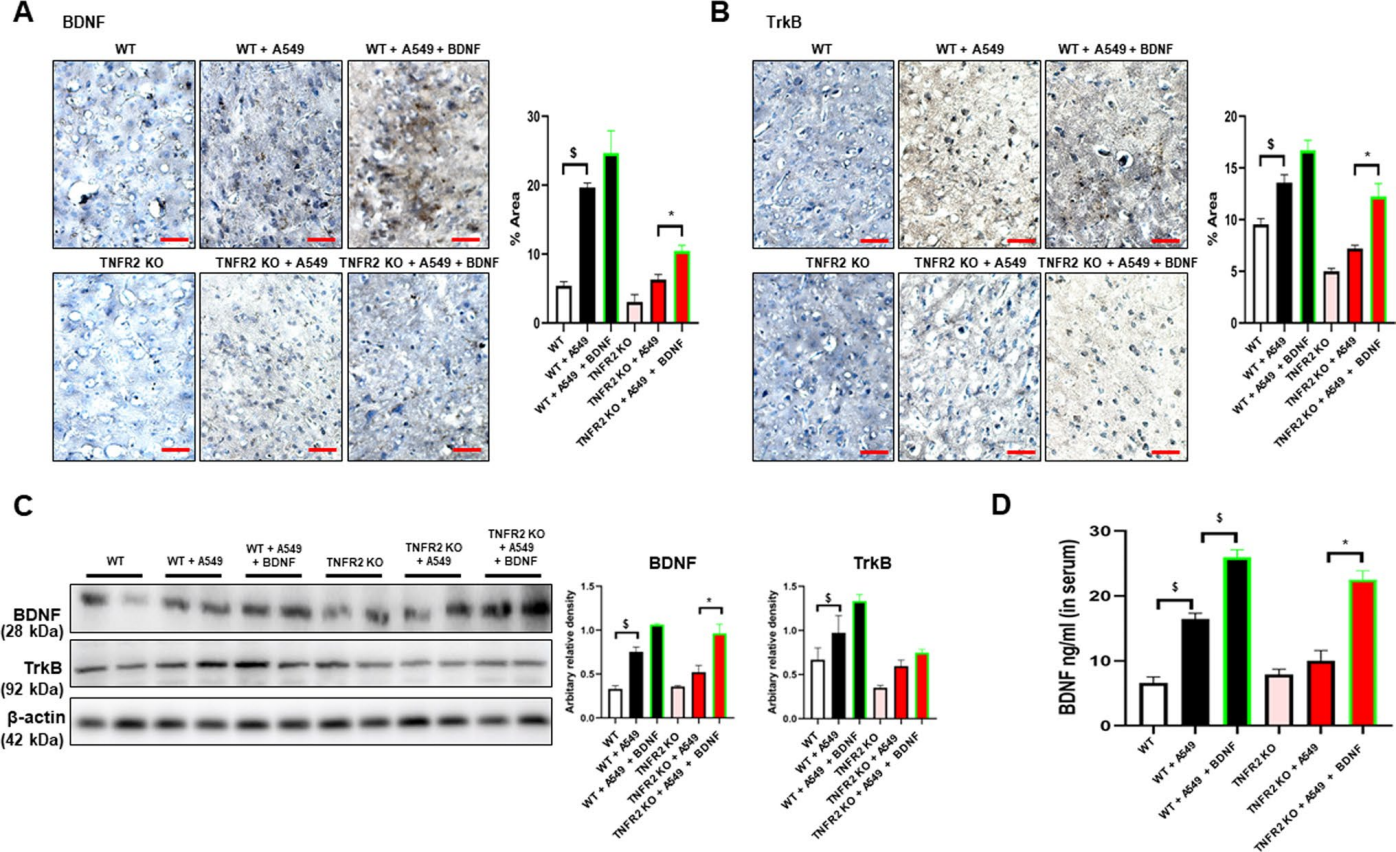
Significant effect on expression level of BDNF and TrkB by BDNF injection in TNFR2 KO xenografted mice brain. Brain sections were analyzed by immunohistochemistry (BDNF; **A**, TrkB; **B**). All slides were counterstained by hematoxylin. Immunohistochemistry was repeated from three independent experiments. Scale bars represent 100 μm. Brain extracts were analyzed by Western blotting. Samples (20 μg) were resolved on 12% SDS–PAGE and detected with antibodies against BDNF, TrkB, and β-actin (**C**). β-actin was internal control. The values on the Western blot bands represent the arbitrary density measured by ImageJ. Values are means ± SEM. *p < 0.05 compared with the TNFR2 KO + A549 mice. The protein levels of BDNF were detected by ELISA in mice serum (**D**). *p < 0.05compared with the TNFR2 KO + A549 mice. ^$^p < 0.05, compared with the WT + A549 mice

**Fig. 10:**
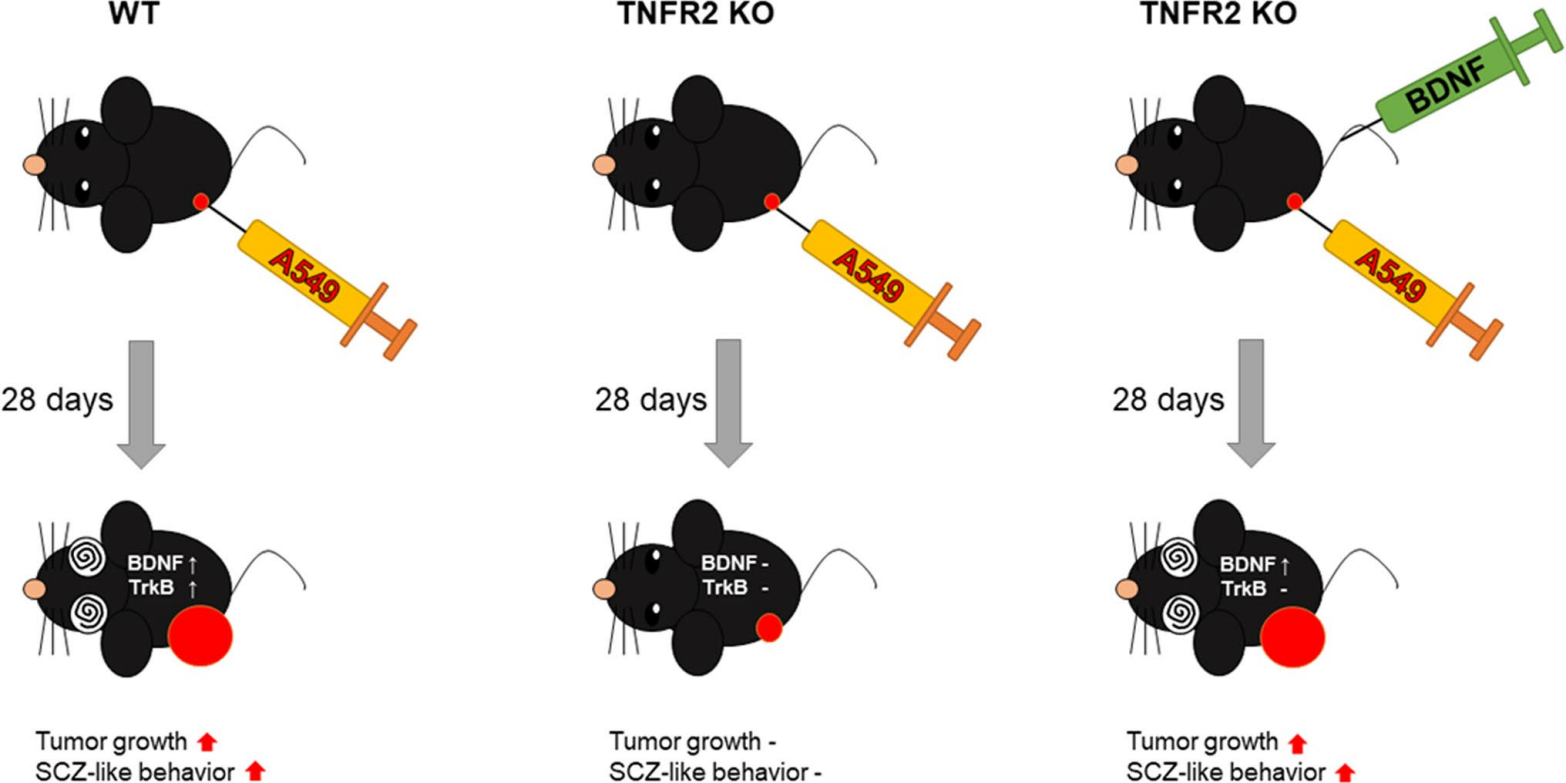
Relationship between TNFR2 KO mice, tumor growth, and schizophrenia

We examined the BDNF and TrkB expression in BDNF-injected TNFR2 KO mice xenografted with A549 lung cancer cells PFC. The expression of BDNF and TrkB detected by IHC in PFC of TNFR2 KO and TNFR2 KO mice xenografted with A549 lung cancer cells was not significant, whereas in WT and WT mice xenografted with A549 lung cancer cells a significant difference was noted. BDNF injection significantly increased BDNF and TrkB expression in the PFC of TNFR2 KO mice xenografted with A549 lung cancer cells, whereas there was no significant difference in the PFC of WT mice xenografted with A549 lung cancer cells (Fig. 9A and B). The expression of BDNF detected by Western blotting was significantly increased in BDNF-injected TNFR2 KO mice xenografted with A549 lung cancer cells, but no significant increase was noted in WT mice xenografted with A549 lung cancer cells. However, the expression of TrkB was not significant (Fig. 9C). The level of BDNF in serum was significantly increased in BDNF-injected TNFR2 KO and WT mice xenografted with A549 lung cancer cells compared to TNFR2 KO and WT mice xenografted with A549 lung cancer cells (Fig. 9D). These results indicate that BDNF injection increases BDNF level systemically, and increases tumor growth and SCZ-like behavior despite TNFR2 KO. Our study is summarized in Fig. 10.

## Discussion

The intricate nature of schizophrenia (SCZ) has been evidenced that SCZ is not a singular disease state and often accompanies with an increased risk of disorders such as dementia, anxiety, or other conditions including cancer (McGinty et al. 2012; Braga et al. 2013; Kodesh et al. 2020; González-Rodríguez et al. 2020). The incidence of heavy smoking was higher in those with psychiatric disorders than that in the general population, and the incidence of lung cancer in SCZ patients was significantly higher than that in the general population (McCreadie 2003; Grinshpoon et al. 2005). Other studies have demonstrated that the risk of cancer was higher in SCZ patients in the specific sites: lung, corpus uteri, and breast (Grinshpoon et al. 2005; Driver 2014). Some cohort studies suggested that SCZ patients have a lower risk of cancer, but an explanation of the underlying mechanism is lacking (Barak et al. 2005; Hippisley-Cox et al. 2007; Xu et al. 2017).

Antipsychotic medications, such as olanzapine, quetiapine, and risperidone, have been suggested to have therapeutic effects in both SCZ and cancer patients by stimulating the immune system (Dalton et al. 2006; Fond et al. 2012; Feigenson et al. 2014; Morozova et al. 2022). Inflammation and immune system dysfunction have been identified as important risk factors for both SCZ and cancer (Kinney et al. 2010; Marballi et al. 2010). Additionally, various factors, including cytokines (interferon, interleukin, and tumor necrosis factor), neurotransmitters (dopamine, GABA, and glutamate), and growth factors, have been proposed to be associated with both SCZ and cancer. However, the underlying mechanisms of the roles of these cytokines linking these two diseases have not been fully understood.

The role of TNFR2 in the development of both SCZ and cancer has been the subject of several studies (Reyes-Gibby et al. 2013; Gough and Myles 2020). It has been suggested that TNFR2 may be a target as important cancer therapeutic (Vanamee and Faustman 2017; Al-Hatamleh et al. 2019). It was found that higher TNFR2 expression in human non-small cell lung cancer leads to a more advanced clinical stage and shorter survival (Zhang et al. 2019). TNFR2 is not only highly expressed in tumor cells but also in immunosuppressive cells, including Treg cells and myeloid-derived suppressor cells (MDSCs) (Sheng et al. 2018). Thus, TNFR2 is considered a tumor-promoting factor and participates in tumor progression by promoting tumor cell proliferation, activating immunosuppressive cells, and supporting immune escape (Al-Hatamleh et al. 2019). In schizophrenia, the transmembrane TNFα is considered a novel target for schizophrenia, and its receptor TNFR2 may be an important factor that mediates immune signaling. Inhibiting transmembrane TNFα signaling by blocking TNFR2 has been reported to attenuate SCZ-like behavior in an animal model (Yeo et al. 2022). Additionally, SCZ patients had increased plasma TNF markers such as TNF, TNFR1, and TNFR2 compared to healthy controls and showed a higher risk of developing SCZ (Hoseth et al. 2017). However, the causal role of TNFR2 in mediating the correlation between SCZ and cancer has not yet been reported.

To explore the potential role of TNFR2 in linking the development of SCZ and cancer, the current study was performed tumor growth and SCZ-like behavioral tests in TNFR2 knockout (KO) mice xenografted with lung cancer cells (A549). Our data showed that the absence of TNFR2 reduced SCZ-like behavior in A549 lung cancer cells xenografted TNFR2 KO mice. The tumor volume and weight of xenograft A549 lung cancer cells were also significantly decreased in TNFR2 KO mice compared to WT mice. Experiments on TNFR1 KO mice were also conducted at the same time, but overall significance was found to be weaker compared to that of TNFR2 KO mice. Our gene-disease association analysis using the discovery platform showed that SCZ was associated with several cancers (Table 1), and TNFR2 could be a common target for the development of two diseases (Tables 2 and 3). The risk of cancer was higher in SCZ than in the general population in the following specific sites: lung in men, and corpus uteri and breast in women (Grinshpoon et al. 2005; Wootten et al. 2022). Gene–gene or protein–protein association, analysis showed that TNFR2 was associated with LTA and ADAM17. In a previous study, LTA was found to be related to SCZ in genetic polymorphism variant (Arab and Elhawary 2015), and ADAM17 was identified as an emerging therapeutic target for lung cancer and its expression was revealed SCZ through the TNF pathway (Hoseth et al. 2017; Saad et al. 2019). Behavioral defects related to SCZ symptoms were related to tumor volume evidenced by the showing fewer behavioral defects due to smaller tumor sizes compared to WT mice. As there were no significant differences in the overall distances measured by OFT, the part which is related to sickness behavior. Especially, the behavioral patterns associated to negative symptoms showed a noteworthy correlation with the size of the tumor. Thus, there exists a possibility that TNFR2 could be a main target for the development of both lung tumor growth and SCZ. However, the mechanisms by which TNFR2 deficiency reduces both lung tumor growth and SCZ symptoms remain unclear.

Furthermore, the study investigated the role of BDNF and its receptor TrkB in mediating the development of both SCZ and cancer. BDNF, a crucial factor in various brain processes, has been studied as a marker of neuropsychiatric diseases and has been linked to cognitive impairment in neurological disorders (Feigenson et al. 2014; Morozova et al. 2022). Additionally, BDNF has been implicated in the pathophysiology of SCZ, with increased serum levels observed in chronic institutionalized patients with schizophrenia (Reis et al. 2008). Recent reports also suggest that BDNF/TrkB pathway plays a role in neuronal tumors, such as neuroblastoma, and may have oncogenic effects in various cancers, including lung, breast, and prostate (Yin et al. 2015; Zhang et al. 2016; Radin and Patel 2017). In the case of non-small cell lung cancer, BDNF treatment has been shown to counteract cisplatin-induced cell death and reduce the chemosensitivity of neuroblastoma cells to cisplatin, with a role of PI3K implicated in BDNF's effects (Ray et al. 2022). BDNF expression was related to ADAM17 through the TNF pathway (Kommaddi et al. 2011). A previous study has highlighted the significance of BDNF expression in the prefrontal cortex (PFC) of individuals with schizophrenia (Weickert et al. 2003). Therefore, we focused on the PFC BDNF level of mice, identifying its potential relevance to the observed phenomena related to SCZ. It was reported that increase of BDNF attenuates SCZ (Grillo et al. 2007), but in some studies, there is also a report that an increase in BDNF rather increases the negative symptoms of SCZ (Reis et al. 2008; Manning et al. 2016). In our study, the expression levels of BDNF/TrkB were significantly lower levels in TNFR2 KO mice xenografted with A549 lung cancer cells compared to WT mice which showed lower development of cancer and SCZ. However, BDNF levels through i.v. injection was increased in TNFR2 KO mice, and this injection resulted in tumor growth and SCZ-like behavior (especially negative symptoms), accompanied with increased BDNF levels. While the levels of the BDNF receptor TrkB remained unaffected at TNFR2 KO mice xenografted with A549 lung cancer cells.

Overall, the results suggest that TNFR2 may be a key mediator in the development of both SCZ and cancer, possibly through the regulation of BDNF expression. In conclusion, this study provides evidence of a possible correlation between the development of cancer and SCZ-like behaviors and suggests that TNFR2 and its effects on BDNF expression could be important in the mediating the relationship between cancer and SCZ development.

### Supplementary Information

Below is the link to the electronic supplementary material.

Supplementary file1 (DOCX 357 kb)

## Data Availability

The data used and/or analysed during the current study are available from the corresponding authors on reasonable request.
