# Supplementary material for: TNF receptor 2 knockout mouse had reduced lung cancer growth and schizophrenia-like behavior through a decrease in TrkB-dependent BDNF level
Source: Arch Pharm Res. 2024 Apr 9;47(4):341–59. doi: 10.1007/s12272-024-01487-0 (PMC11045614; doi:10.1007/s12272-024-01487-0)
Supplement: Supplementary file 1 — Supplementary file1 (DOCX 357 kb) [file 12272_2024_1487_MOESM1_ESM.docx]

**Supplementary figure**

**
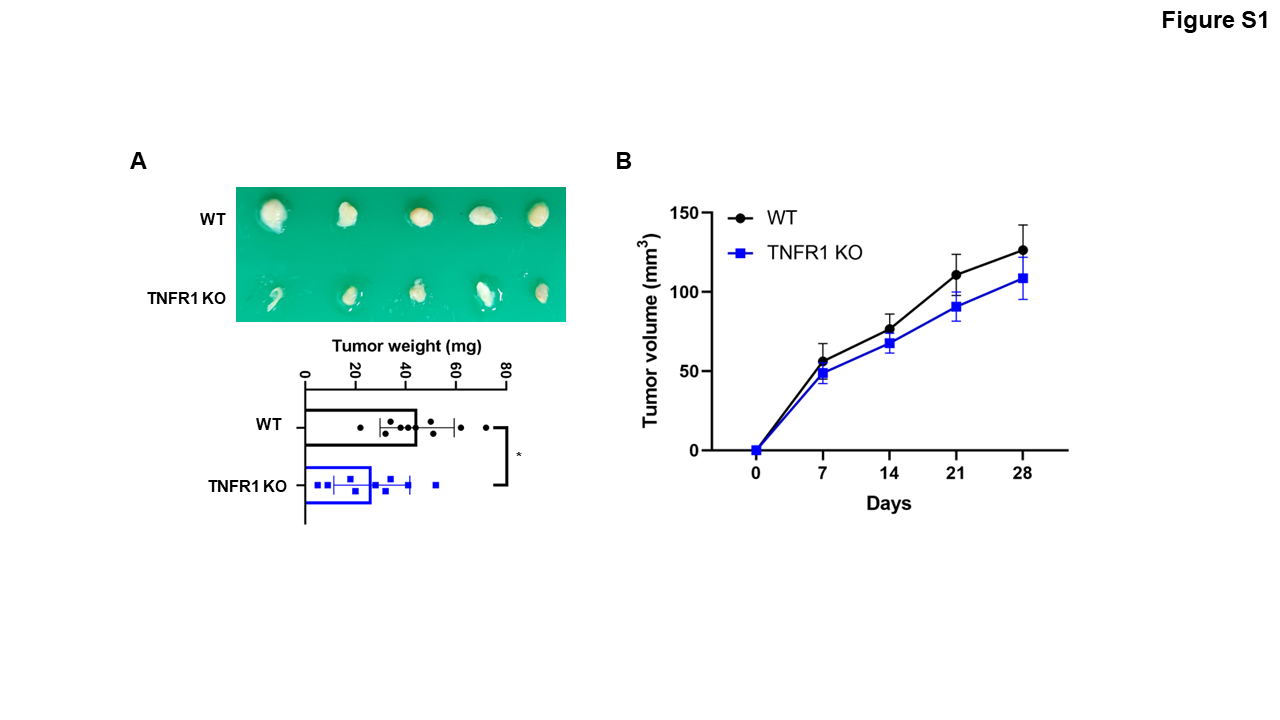
**

**Figure S1. Tumor growth in TNFR1 KO mice.**

Cancer cells (A549) were injected into the right lower flank in mice. All data indicate tumors formed on the right lower flank. Tumor (A) volumes and (B) weights. Points and columns, means of animals (WT + A549; n=10, TNFR1 KO + A549; n=9). Values are means ± SEM. *p<0.05 compared with the WT mice.

**
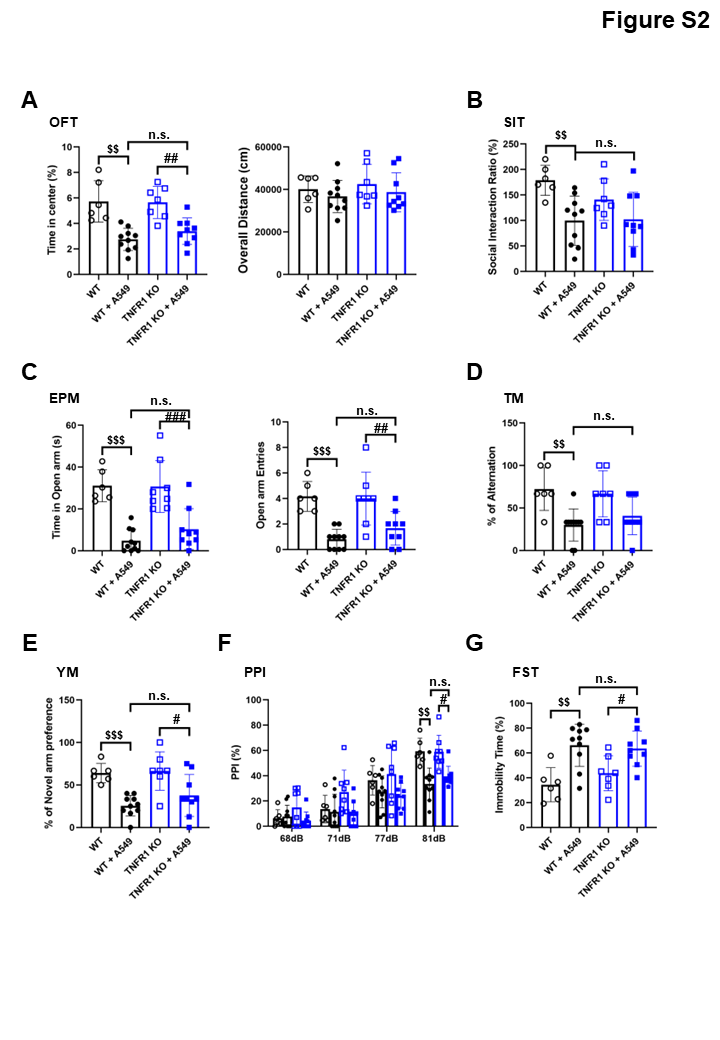
**

**Figure S2. Effects on SCZ-like behavior by xenograft in TNFR1 KO.**

Locomotor activity and anxiety measured as time in central zone (%) and overall distance (cm) in open field test (OFT) (A). Social anxiety disorder was measured as time in interaction zone (%) in social interaction test (SIT) (B). Basal anxiety assessed as time (in sec) and entries in open arms in the elevated plus maze (EPM) (C). Cognitive impairment was measured by T-maze (TM) (D) and Y-maze (YM) (E). Sensorimotor gating was measured by pre-pulse inhibition test (PPI) (F). Depression immobility time (in sec) in forced swimming test (FST) (G). Data are represented as means ± SEM (WT; n=6, WT + A549; n=10, TNFR1 KO; n=7, and TNFR1 KO + A549; n=9). ^$$^p<0.005, ^$$$^p<0.001 compared with the WT +A549 mice. ^#^p<0.05, ^##^p<0.005, ^###^p<0.001 compared with the TNFR1 KO + A549 mice.
